# Supplementary material for: Whole genome SNP typing to investigate methicillin-resistant Staphylococcus aureus carriage in a health-care provider as the source of multiple surgical site infections
Source: Hereditas. 2016 Nov 14;153:11. doi: 10.1186/s41065-016-0017-x (PMC5226111; doi:10.1186/s41065-016-0017-x)
Supplement: Additional file 1: — Table S1. Sequencing quality and coverage. Quality breadth is based on the total reference size, 2,780,693 bases, after duplicated regions were removed. In order to pass quality filtering, the loci had to have >10× coverage and a SNP state had to be in >90 % of the reads to be considered. Mean coverage was calculated using a genome size of 2.8 Mb. (DOC 54 kb) [file 41065_2016_17_MOESM1_ESM.doc]

Table S1 Sequencing quality and coverage of each isolate.

| **Patient** | **Quality Breadth** | **% Quality Breadth** | **Mean Coverage** | **No. reads (per fastq file)** |
| --- | --- | --- | --- | --- |
| Surgical Team Member | 2,596,550 | 89.00% | 102 | 1,417,881 |
| Case 2 | 2,594,326 | 88.92% | 97 | 1,353,234 |
| Case 3a | 2,739,135 | 93.89% | 90 | 1,255,348 |
| Case 3b | 2,738,329 | 93.86% | 63 | 879,623 |
| Case 3c | 2,737,954 | 93.85% | 65 | 904,904 |
| Case 4 | 2,600,313 | 89.13% | 92 | 1,282,996 |
| Hospital Control 1 | 2,589,579 | 88.76% | 106 | 1,475,900 |
| Hospital Control 2 | 2,554,780 | 87.57% | 93 | 1,300,233 |
| Hospital Control 3 | 2,592,863 | 88.87% | 80 | 1,117,302 |
| Hospital Control 4a | 2,587,831 | 88.70% | 75 | 1,051,604 |
| Hospital Control 4b | 2,589,447 | 88.76% | 74 | 1,027,736 |
| Hospital Control 5 | 2,742,098 | 93.99% | 106 | 1,480,472 |
| Hospital Control 6 | 2,595,027 | 88.95% | 88 | 1,221,490 |
| Hospital Control 7 | 2,742,098 | 94.00% | 234 | 3,244,524 |
| Hospital Control 8a | 2,591,126 | 88.81% | 148 | 2,063,015 |
| Hospital Control 8b | 2,589,391 | 88.75% | 204 | 2,834,116 |
| Healthcare Control 9a | 2,742,288 | 94.00% | 83 | 1,159,349 |
| Healthcare Control 9b | 2,741,988 | 93.99% | 186 | 2,578,711 |
| Healthcare Control 10 | 2,592,699 | 88.87% | 156 | 2,162,667 |
| Healthcare Control 11 | 2,742,264 | 93.99% | 110 | 1,534,436 |
| Community Control 12 | 2,741,909 | 93.98% | 75 | 1,043,995 |
| Community Control 13 | 2,742,104 | 93.99% | 64 | 888,222 |
| Community Control 14 | 2,742,324 | 94.00% | 74 | 1,029,049 |
| Community Control 15 | 2,740,004 | 93.92% | 40 | 561,702 |
