# Supplementary material for: Whole genome SNP typing to investigate methicillin-resistant Staphylococcus aureus carriage in a health-care provider as the source of multiple surgical site infections
Source: Hereditas. 2016 Nov 14;153:11. doi: 10.1186/s41065-016-0017-x (PMC5226111; doi:10.1186/s41065-016-0017-x)
Supplement: Additional file 2: — Table S2. Multi-Locus Sequence Type results obtained from WGS data using SRST2. NF indicates not found and (*) indicates mismatches. (DOC 52 kb) [file 41065_2016_17_MOESM2_ESM.doc]

Table S2 Multi-Locus Sequence Type results obtained from WGS data using SRST2. NF denotes not found. (*) denotes mismatches.

| **Sample** | **SequenceType** | **arcC** | **aroE** | **glpF** | **gmk** | **pta** | **Tpi** | **yqil** |
| --- | --- | --- | --- | --- | --- | --- | --- | --- |
| Surgical Team Member | 225 | 1 | 4 | 1 | 4 | 12 | 25 | 10 |
| Case 2 | 105 | 1 | 4 | 1 | 4 | 12 | 1 | 28 |
| Case 3a | 8 | 3 | 3 | 1 | 1 | 4 | 4 | 3 |
| Case 3b | 8 | 3 | 3 | 1 | 1 | 4 | 4 | 3 |
| Case 3c | 8 | 3 | 3 | 1 | 1 | 4 | 4 | 3 |
| Case 4 | 5 | 1 | 4 | 1 | 4 | 12 | 1 | 10 |
| Hospital Control 1 | 5 | 1 | 4 | 1 | 4 | 12 | 1 | 10 |
| Hospital Control 2 | 5 | 1 | 4 | 1 | 4 | 12 | 1 | 10 |
| Hospital Control 3 | 5 | 1 | 4 | 1 | 4 | 12 | 1 | 10 |
| Hospital Control 4a | 5 | 1 | 4 | 1 | 4 | 12 | 1 | 10 |
| Hospital Control 4b | 5 | 1 | 4 | 1 | 4 | 12 | 1 | 10 |
| Hospital Control 5 | 8 | 3 | 3 | 1 | 1 | 4 | 4 | 3 |
| Hospital Control 6 | 5 | 1 | 4 | 1 | 4 | 12 | 1 | 10 |
| Hospital Control 7 | 8 | 3 | 3 | 1 | 1 | 4 | 4 | 3 |
| Hospital Control 8a | 5 | 1 | 4 | 1 | 4 | 12 | 1 | 10 |
| Hospital Control 8b | 5 | 1 | 4 | 1 | 4 | 12 | 1 | 10 |
| Healthcare Control 9a | NF* | 3 | 3 | 218* | 1 | 4 | 4 | 3 |
| Healthcare Control 9b | 8 | 3 | 3 | 1 | 1 | 4 | 4 | 3 |
| Healthcare Control 10 | 5 | 1 | 4 | 1 | 4 | 12 | 1 | 10 |
| Healthcare Control 11 | 8 | 3 | 3 | 1 | 1 | 4 | 4 | 3 |
| Community Control 12 | 8 | 3 | 3 | 1 | 1 | 4 | 4 | 3 |
| Community Control 13 | 8 | 3 | 3 | 1 | 1 | 4 | 4 | 3 |
| Community Control 14 | 8 | 3 | 3 | 1 | 1 | 4 | 4 | 3 |
| Community Control 15 | 8 | 3 | 3 | 1 | 1 | 4 | 4 | 3 |
